# Company-Level Family Policies: Who Has Access to It and What Are Some of Its Outcomes?

**DOI:** 10.1007/978-3-030-54618-2_21

**Published:** 2020-07-16

**Authors:** Heejung Chung

**Affiliations:** 1 Swedish Institute for Social Research (SOFI), Stockholm University, Stockholm, Sweden; 2 Centre for Sociological Research, University of Leuven, Leuven, Belgium; University of Kent, Canterbury, UK

## Abstract

Despite the increase in the number of studies that examine the cross-national variation in the policy configuration that allow a better work–family integration, very few look beyond the national levels. It is also crucial to examine occupational level welfare since companies may restrict or expand the existing national-level regulations, defining the “final availability” workers actually have toward various arrangements. In addition, companies may provide various additional arrangements through occupational policies which are not set out in the national-level agreements that are crucial in addressing reconciliation needs of workers. This chapter examines what types of arrangements are provided at the company level to address work–family demands of workers. It further provides a synthesis of studies that examine both national-level contexts and individual-level characteristics that explain who gets access to company-level family-friendly policies, which is linked to the possible outcomes of these policies.

Most industrial societies have seen a rise in women taking part in the labor market in the past two decades (Chung & Van der Horst, 2018b). With it, we also observe changes in gender norms in whose role it is to care for children and elderly/disabled family member, and whose role it is to do the breadwinning (Knight & Brinton, 2017). Increasing numbers of men and fathers are voicing their interest in taking a larger part in childcare (Working Families, 2017), and with it, there is a rise in the demand from workers for a better work–life balance and demand for more family-friendly policies at the company level. For example, studies have shown that there are more workers who consider work–life balance as (very) important when considering their next job compared to those who believe other more traditional factors such as higher income is important (Chung, 2017). More recent studies have also shown that many workers place flexible working, one of the most common types of family-friendly arrangement currently used across Europe and the US, as the top benefit they would like in the workplace exceeding in many cases other more financial types of benefits (Franklin, 2019; Scott, 2018). This demand is more prevalent among millennials—i.e., those born between 1983 and 1995 (Deloitte, 2018). Some studies have shown that 4 out of 10 millennials have said they have refused a job due to the lack of flexibility (Franklin, 2019), compared to a quarter for all workers.

This chapter aims to closely examine who has access to company-level family policies and what its outcomes are for the company, the individual, their family, and possibly society as whole. This is done through synthesizing a range of studies that have examined company-level family policies focusing mostly on studies in Europe, but with references to studies that have been carried out in the US and other countries. Although it aims to capture a wide range of family policies, many of the studies examined focus on working-time flexibility arrangements—a frequented topic of study given the increased demands for such arrangements.

The next section defines what we mean by family-friendly policies at the company level, followed by an examination of some of the trends of family policies using some secondary quantitative data. The next section provides some key summaries of the outcomes of family-friendly policies at the company level. This includes outcomes for the worker, their family, and the company. The final section sums the chapter up, with some final thoughts on the frontiers of research in the field and what needs to be done to develop the field in the future.

## Defining Family-Friendly Policies and Flexible Working

Family-friendly policies can be defined as policies that directly support the combination of professional, family, and private life (Plantenga & Remery, 2005). Company-level policies are those introduced or implemented by firms to enhance work–life balance of workers. This does not have to be the firm’s independent policy and could involve the implementation of national or sectoral collective agreements or legal regulations. Companies can restrict access to policies that are implemented at the national and sectoral level so that de facto workers are not able to take them up. On the other hand, companies can provide additional policies that do not exist at the national or sectoral levels to help workers balance work with other aspects of life to meet a range of different needs that companies themselves face (Chung, 2012; Chung & Tijdens, 2013). This is why many studies find a discrepancy between the national and company-level practices in relation to family-friendly policies (Den Dulk, 2001; Ollier-Malaterre, 2009), and why sometimes scholars define company-level policies as the “ final availability” workers actually have toward various arrangements (Chung & Tijdens, 2013; Lambert & Haley-Lock, 2004). Having said this, sometimes this availability written in company policies is also not a guarantee that workers feel comfortable taking them up (Cooper & Baird, 2015). This is especially the case when there is a culture within the organization and the profession which stigmatizes workers who take up family-friendly working arrangements—i.e., the so-called “ flexibility stigma” (Chung, 2018b; Williams, Blair-Loy, & Berdahl, 2013), which can hinder the take-up of arrangements even when they are available.

There are several different types of family-friendly arrangements that are commonly provided by companies, and examined by work–family scholars.

First, there are family-friendly working-time arrangements, or what others call employee-friendly working-time arrangements (Chung & Tijdens, 2013; Rubery & Grimshaw, 2003). This includes arrangements that allow workers to have more control over when they work—i.e., *flexitime* (flexible starting and ending time of work), condensed or compressed working hours (for example, working full-time over four rather than five days), annualized hours (where working time is calculated not over the course of the week but across a longer period of time—up to the whole calendar year), working-time autonomy (where workers have almost complete control over when and how much they work, as long as the work gets done), or the ability to take a couple of hours off work to tend to personal issues. Family-friendly working-time arrangements also include arrangements that allow changes in the hours worked by the worker, that is in most cases the ability to work less than full-time, albeit sometimes temporarily, to fit workers’ needs to balance family or life with work. This includes part-time working (namely, working less than full-time, in some cases defined as working less than 30 hours a week), term-time working (where workers work only during school term times), temporary reduction of hours (where workers work reduced hours for a short period of time), and phased retirement (where workers gradually reduce the number of hours of work before retirement). Although not directly related, many group these family-friendly working-time arrangements with other types of arrangements such as teleworking/ home working to brand them as flexible working arrangements or schedule control (Dex & Scheibl, 2001; Glass & Estes, 1997; Kelly, Moen, & Tranby, 2011; Lewis & Humbert, 2010).

Second, there are arrangements provided by companies in which workers take a longer period of time off work to take care of their responsibilities outside of work, such as maternity/paternity, parental, and carer’s leave. Companies can either provide additional time off, or provide top-up of benefits given during this period. For example, a large number of companies in Sweden provide additional parental leave pay which tops up the benefit levels set by the national policies (Duvander & Löfgren, 2019). In a broader perspective of work–life balance, these types of arrangements can also include (paid) leave for education, training, and general sabbaticals.

Third, there are services provided by the company. These can be in the form of facilities, such as kindergarten or other childcare amenities including in-house crèches. Or they can be in the form of financial support, for example, for parents using private childcare facilities or other care support services. This can also include support for other types of household work—e.g., laundry facilities.

## Provision of Family-Friendly Arrangements Across Europe

There is not a lot of cross-nationally comparative data on the extent to which this wide range of arrangements is provided in companies. One of the few existing surveys that cover a wide range of arrangements and is comparable across Europe is the Establishment Survey for Working Time and Work–Life Balance (ESWT). The ESWT covers establishments of 10 or more employees across 21 European countries and was collected in 2004/5 (for more, see Riedmann, Bielenski, Szczurowska, & Wagner, 2006). The more recent version of this survey, the European Company Survey, does provide information on some working-time arrangements—e.g., flexitime provision—yet does not cover information about family-friendly leaves or services provided by the company. This raises a serious issue about the lack of comparable data sets on company-level provisions of family-friendly policies which needs to be addressed. Chapter 10.1007/978-3-030-54618-2_22 by Begall and Van der Lippe in this volume details one such innovation.

According to the Establishment Survey for Working Time and Work–Life Balance, 48% of all companies reported providing some sort of flexitime arrangements for their workers in 2004/5. Note that this number has increased to 57% in 2009, and 65% in 2013 according to the European Company Survey data (Chung, 2014). In the 2004/5 survey, 53% of all companies said they provided some sort of leave options, 37% for care of elderly care, ill or disabled relatives, 36% provided leave for further education, and 26% for other purposes (excluding parental leave). Examining the childcare facilities offered by establishments, approximately 3% of all establishments offered an own company kindergarten or crèche services, while 2% offered other forms of childcare help—with larger companies and companies within the service sector more likely to offer such services (Riedmann et al., 2006). There are large variations across countries in the extent to which services and leaves are provided. Chung (2008b) examines the cross-national variation in the diversity of arrangements provided at the establishment levels using the following operationalization based on the ESWT data:

- **Work–life balance through working time** (4 options) = use of part-time work in the employee’s interest + possibility to change from full-time to part-time on request +  flexitime used in the employee’s interest + working-time accounts in the employee’s interest
- **Work–life balance through leaves** (4 options) = parental leave + leave for care + leave for education + leave for other purposes
- **Work–life balance through** **services** (4 options) = use of kindergarten or crèche + help for childcare + help for household management + other services
- **Total work–life balance option provision** (12 options) = working-time score + leave score + services score

**Fig. 21.1:**
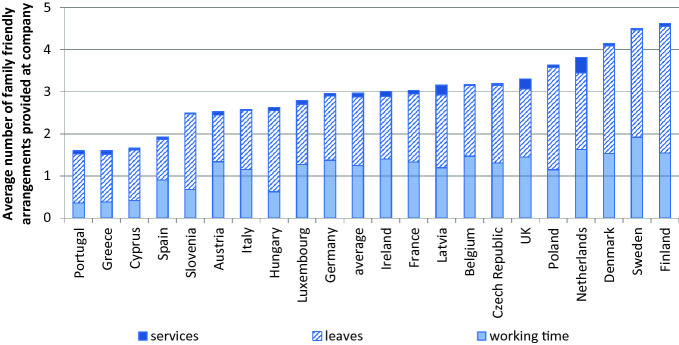
Cross-country variation in the provision of family-friendly arrangements for 21 European countries (establishment weighted) (*N* = 17,308) (*Source* Chung, 2008b)

As shown in Fig. 21.1, leaves and working-time arrangements were the most commonly provided family-friendly arrangements while not many establishments provide services to their workers. Examining the cross-national variation of the company-level policies, we see that the Northern European countries Finland, Sweden, and Denmark, which are typically known to have generous national-level family policies (Bettio & Plantenga, 2004; Ferragina & Seeleib-Kaiser, 2015; Korpi, Ferrarini, & Englund, 2013; Plantenga & Remery, 2009) are the forerunners in the provision of family-friendly arrangements. This is also the case in the Netherlands, well known for its flexible labor market (Wilthagen & Tros, 2004). On the other hand, Southern European countries—namely, Portugal, Greece, Cyprus, and Spain—are those where establishments do not offer much in terms of family-friendly arrangements.

When examining more recent data, focusing on the access to flexible working arrangements using individual-level data, similar patterns are observed. Based on the most recent European Working Conditions Survey of 2015, Figs. 21.2, 21.3 and 21.4 show the extent to which workers have access to a number of family-friendly working-time arrangements. Due to lack of data, we are unable to look at workers’ perceived access to other types of family-friendly arrangements. Flexitime is defined in the Working Conditions Survey as a worker being able to “adapt their working hours within certain limits,” while working-time autonomy is defined as a job “where your working hours are entirely determined by yourself.” Time off work for personal reasons include those who have answered “very easy” or “fairly easy” to the question “Would you say that for you arranging to take an hour or two off during working hours to take care of personal or family matters is…”. Those who work from home are defined here as those who have worked in their home at least several times a month in the past 12 months, and this group and those who have worked in public spaces at least several times a month in the past 12 months are considered those who teleworked.

**Fig. 21.2:**
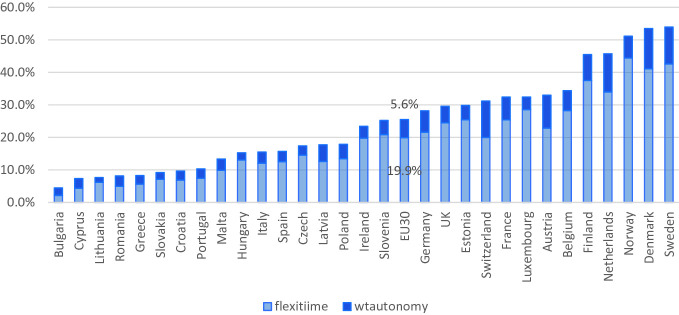
Proportion of dependent employed with schedule control across 30 European countries in 2015 (*Source* EWCS, 2015; Chung, 2019a)

**Fig. 21.3:**
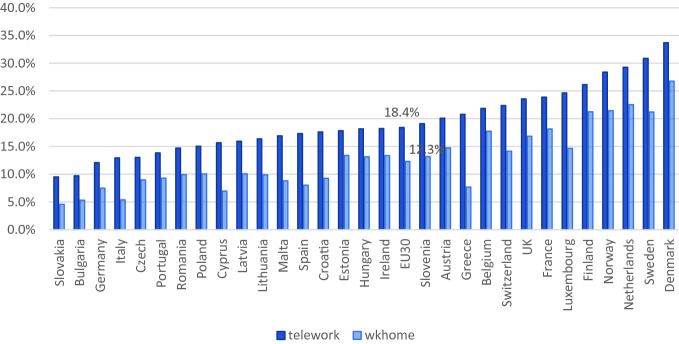
Proportion of dependent employed who have worked at home or in public spaces several times a month in the past 12 months across 30 European countries in 2015 (*Source* EWCS, 2015; Chung & van der Lippe, 2018)

**Fig. 21.4:**
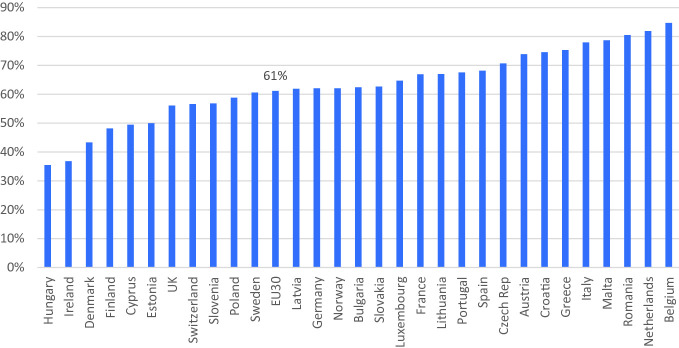
Proportion of dependent employees across 30 European countries with access to time off during working hours for personal reasons in 2015 (*Source* EWCS, 2015, author’s calculations)

Figure 21.2 shows more than a quarter of all dependent employed workers across the 30 European countries, that is the 28 member states plus Switzerland and Norway, have some sort of schedule control. Approximately 20% have access to flexitime and another 6% full working-time autonomy. There is a clear pattern here, again with Northern European countries—such as Sweden, Denmark, Finland, and the Netherlands—being the ones where flexitime and working-time autonomy is prevalent. On the other hand, Eastern and Southern European countries, which also have limited family-friendly policies at the national level, are the ones where access to family-friendly working-time arrangements are also limited. Similar patterns emerge when examining the patterns of teleworking across Europe, wherein the Northern European countries workers are more likely to have worked at home or in other public spaces regularly in the past 12 months. On the other hand, Southern and Eastern European countries are those where such patterns of work are not as prevalent. Actually, previous studies have shown that in countries where national family policies are generous, workers are also more likely to have access to company-level family-friendly working-time arrangements as well (Chung, 2018a, 2019a; Den Dulk, Groeneveld, Ollier-Malaterre, & Valcour, 2013). This pattern is not as clear-cut in the case for time off work for personal reasons, as shown in Fig. 21.4. First of all, it is noticeable how widespread this arrangement is in comparison to the other types of arrangements, with 61% of all dependent employees noting that they are able to take a couple of hours off work to tend to personal/family issues. Although the Netherlands and Belgium remain in the top group of countries, where workers note that these arrangements are accessible, other countries with the highest level of provision include Romania, Malta, and Italy, all of which were not forerunners in terms of the provision of other types of family-friendly working-time arrangements. This could possibly be due to the fact that in the case where workers have greater flexibility in their schedule, they can try to tend to family and other personal issues outside of working hours. On the other hand, those who are more fixed to a 9–5 schedule within the office, may have to resort to taking a couple of hours off work during working hours to manage their personal/family issue. Further investigation is needed.

We expect that there will be an increase in the use of family-friendly working-time arrangements in Europe in the future due to the mass scale home working that was encouraged or enforced during the COVID-19 lockdown periods across all countries (Chung et al., 2020). In addition, there is likely to be a rise in flexible working due to the new European Directive on Work–Life Balance that has been passed by the European parliament in 2019.^1^ The new directive includes the right to request flexible working for parents of children and workers with care responsibilities, which provides workers across Europe a stronger right to access flexible working arrangements—namely, what we discussed above as family-friendly working-time arrangements and workers’ ability to work from home. Although the directive aims to influence national-level legislations, family-friendly working-time arrangements are de facto provided at the company level. In this sense, wide spread of national-level legislation that provides workers the right to request flexible working is expected to shape company-level provisions and accordingly workers’ access to these arrangements. Given that many European countries—such as the UK, the Netherlands, Finland, and Italy—already have some legal provision similar to this already, the larger changes will occur in other countries where no such rights exist at the moment. However, more research is needed to investigate this further.

## Who Has Access to Family-Friendly Arrangements?

### National-Level Determinants^2^

In this section, we turn to national-level determinants of access to family-friendly policies—again focusing more specifically on flexible working arrangements. Table 21.1 reviews 18 existing studies that examine the use of family-friendly policies, or flexible working arrangements in a cross-national perspective. From the table we can see that, industrial relations and power resources of unions, cultural factors including national norms on gender issues and work orientation, the institutional factors, i.e., family policies, national-level demand, measured through women’s labor market participation, and economic conditions and structures, i.e., affluence of the country, economic labor market condition, the composition of the economy, are all relevant factors we can consider when examining the national-level factors that explain who provides more family-friendly arrangements.

**Table 21.1:** Review of 18 existing cross-national study on family-friendly arrangements/flexible working arrangements

	Study	Dependent variable	Data	Country level determinants of provision of flexitime
Interviews with managers and others	Den Dulk (2001, 2005)	Family-friendly arrangements including flexitime	Interviews with HR officers of service sector in NL, IT, UK, SE (1998–1999)	Employers in liberal countries use family-friendly policies as retention policies (so a negative relationship between policy and company level provision—extra provision)
	Berg et al. (2004)	Working time, flexible work schedules, and employee control over working time	Interviews with managers of 7 different countries DE, SE, NL, IT, JP, AUS, US (2000)	Collective bargaining coverage, high trade union density, representatives who were sensitive toward working time issues, workers have more control over working time (Germany, Sweden, the Netherlands)—in liberal countries, the high-skilled professionals have more control, in these countries flexible form of working is narrow
	Ollier-Malaterre (2009)	Provision of family-friendly policies	44 in depth semi-structured interviews across 16 organizations in France of HR managers, employee representatives, diversity officers etc. (2005–2006)	Companies in countries without statutory regulations may use family-friendly arrangements more
	Berg, Kossek, Baird, and Block (2013)	Flexible scheduling, vacation leave and parental leave	Interviews conducted with managers, supervisors, and labor union representatives from two universities each in US, AUS (2006–2008)	Flexitime provided only through employer’s discretion in both cases, but universities with more unified bargaining structure—single table agreement reduced the likelihood of employers whipsawing the request for flexible work
Individual level data	Evans (2001, 2002)	Various types of family-friendly arrangements including flexitime	European Working Conditions Survey 15 European countries (1995)	Impact of national level policies and extra statutory maternity leave company policy is not clear cut—U-shaped—state provision crowds out company level provision only at a very high level—bivariate analysis
	Lyness et al. (2012)	1. Control over work schedule starting and stopping times (flexitime) 2. Control over the number of hours worked	International Social Survey Programme 21 countries (1997)	Control over work related to GDP per capita (+), social policy expenditure (+), collective bargaining coverage (+), and paid leave policies (+) (variables included: GDP cap, Social Exp, Women’s LF part, Service Sector emp, Union Cov, Weekly Hours Policy (collective agreed hours), Paid Leave Policy)—ML analysis
	Ortega (2009)	Employee discretion (they can choose the order, the method, the speed or rate of work, the timing of breaks, or the working hours) summative index	EWCS 15 EU countries (2000)	Employee discretion—control over one’s working hours is stronger in countries with higher female labor market participation rates—Multivariate analysis one country characteristic
	Plantenga and Remery (2010)	Flexitime, working time banking, staggered working hours	EU-LFS Reconciliation between Work and Family Life 29 EU countries (2004)	Nordic countries more likely to use flexitime/working time banking, Eastern European, Southern European countries less likely (no context factors examined)—descriptive
	Präg and Mills (2014)	Flexitime and working time banking	EU-LFS Reconciliation between Work and Family Life 29 EU countries (2010)	GDP per capita (+), social policy expenditure (+), national policies on leaves for care of sick children and adults (- but weak), the female labor force participation (LFP) rate (+), the size of the service sector (+), collective bargaining coverage (+), and gender occupational segregation (n.s.)—bivariate analysis
	Chung (2018a)	Flexitime and time off for personal reasons	EWCS 30 European countries (2015)—women with care responsibilities	Union density (+), collective bargaining coverage (+), public expenditure on family policies % of GDP (+), proportion of children in formal childcare 0–3 (+)—also shows that the effect of national variables stronger for high-skilled workers
	Chung (2019a)	Flexitime (schedule control)	EWCS 27 European countries (2010)	Public expenditure on family policies % of GDP (+), proportion of children in formal childcare 0–3 (+), effective parental leave (U-shaped relationship)
Establishment level data	Den Dulk et al. (2012)	(Change & proportion) use of part-time work arrangements, job sharing, flexitime, home-based work and telework	CRANET data (100+ employees), 19 European countries (1999–2000)	Examines regime differences (social democratic, conservative, liberal, formal communist, Mediterranean)—provision in that order--> no evidence of crowing out, countries with more statutory provision provides more arrangements—ML analysis
	Den Dulk et al. (2013)		Establishment Survey on Working Time—21 European countries (2004/5)	
	Chung (2009)	Employee-centered flexibility (combining flexitime, part-time, reduction of working hours, phased retirement & leaves)	Establishment Survey on Working Time—21 European countries (2004/5)	EPL temp (−), Union density (+), size of public sector (+), female labor market participation (+), unemployment average (+), trade (+) (other variables include EPL regular, centralisation of bargaining, foreign direct investment, service sector employment)—ML analysis
	Chung (2008a)	Working time arrangements (part-time, phased retirement, possibility to change from full-time to part-time, Flexitime, working time banking)	EWST—21 European countries (2004/5)	No clear cross-national variance examined—but Nordic countries, conservative countries with more working time arrangements, southern European countries with least—ML analysis
	Chung (2014)	Flexitime provision (provision as a dichotomous variable, proportion of workers covered, ability to take time off in hours, ability to take days off)	European Company Survey 2009 (and a comparison with 2004; some descriptive on 2013)	Provision of flexitime: Union density (+), collective bargaining coverage (+), centralisation of bargaining (n.s.), gender norms (n.s.), work centrality culture (−), family policy expenditure (+), female labor market participation (+), GDP/capita (+), GDP growth rate (n.s.), unemployment rate (n.s.), size of services sector (+), size of public sector (n.s.)

#### Family and Social Policy

Perhaps one of the most widely examined and one of the most interesting factors for scholars is the influence of national-level family- and other social policies in the provision of family-friendly arrangements at the company level. There are two theoretical assumptions held in examining the relationship between national-level policies and provision of (additional) family-friendly policies by the company. Firstly, “crowding out” theory (Etzioni, 1995)—usually used to examine the relationship between welfare states and social capital—argues that generous national-level social policy programs “‘crowd out’ informal caring relations and social networks, as well as familial, communal and occupational systems of self-help and reciprocity” (Van Oorschot & Arts, 2005, p. 6). Based on this theory, countries where generous family policies exist at the national level, companies will not be willing to or may not feel a need to provide occupational policies to address similar issues. The counterargument to this comes from the “crowding in” theory (e.g., Künemund & Rein, 1999; Van Oorschot & Arts, 2005), which argues that it is rather the countries with generous family policies that usually have companies that also provide more and better family-friendly policies at the company level. The theoretical argument is similar to that of institutional theorists, who argue that institutions, laws, and policies may put pressure on organizations to become similar to national institutions (DiMaggio & Powell, 1983). Den Dulk et al. (2013) argue that governments put institutional pressure on organizations to develop work-life arrangements through coercive powers. Work–life-balance-related policy regulations that enforce provision and tax incentives for such policies directly influence company behaviors in these matters. The pressure can also take the form of normative isomorphic pressure, i.e., national‐level policies changing the norm and subsequent public demand for companies to be more family‐friendly (Den Dulk et al., 2013), or mimetic pressure, i.e., where companies imitate or mimic the practices of other (successful) organizations (Been et al., 2017; Davis & Kalleberg, 2006). Institutional theory argues that institutions and bureaucratic systems, laws, and policies put pressure on organizations to become similar through isomorphic processes. Based on this line of reasoning, we can expect company-level family-friendly policies to be more generous and widespread in countries where there are generous family policies.

Previous studies provide evidence for both crowding in and crowding out. There is evidence that show in countries where there aren’t many statutory regulations on family policies, companies use family-friendly policies as retention or other strategic goals (Den Dulk, 2001, 2005; Ollier-Malaterre, 2009) and thus can be more generous. Others argue that there is no clear relationship between statutory regulations and (extra) company provision (Kassinis & Stavrou, 2013; Präg & Mills, 2014), and only when there is a very large involvement from the state, a crowding-out impact can be seen (Evans, 2002). However, increasingly there is more evidence that countries with generous family policies at the national level are those where companies also tend to be more active in providing family-friendly arrangements (e.g., Been et al., 2017; Den Dulk, Peters, & Poutsma, 2012; Den Dulk et al., 2013; Lyness, Gornick, Stone, & Grotto, 2012). More recently, Chung (2018a, 2019a) argues that the type of policy in question matters in examining the relationship between national-level family policies and the provision of family-friendly arrangements at the company level. There are different associations between the provision of company-level family policies with national-level work-reducing policies (leaves) against “work-facilitating” measures (Misra, Budig, & Boeckmann, 2011). Work-facilitating policies—the extent to which the state encourages women’s labor market participation/ dual-earner system, for example, through public childcare provisions—are positively associated with (“crowd in”) access to family-friendly working-time policies (see also, Chung, 2009; Den Dulk et al., 2013; Lyness et al., 2012). In contrast, work-reducing policies “crowd in” only to a certain degree and then “crowd out,” similar to what was found for women’s employment patterns (see, Misra et al., 2011). Finally, scholars have shown that the crowding in/out may be different depending on the types of companies examined—e.g., public vs private sectors (Den Dulk et al., 2013) and types of workers examined—e.g., high- vs low-skilled workers. Chung (2018a, 2019a) shows how the crowding in of national-level policies are especially stronger for high-skilled workers.

#### Industrial Relations

 Industrial relations at the national level have also been seen to have a major influence on the choices managers/companies make in the provision of family policies, and providing workers with control over work. According to the power resource theory, welfare states are shaped by the power that is mobilized by the wage earners, may it be through political parties or through interest organizations such as labor unions (Korpi, 1989). In addition to the direct impact trade unions may have on shaping national policies, when there are strong unions within the company and at the national level, this will lead to a “contagion from the left” (Korpi, 1989, p. 316) influencing the way employers act in providing family-friendly arrangements at the company level. In addition, in the Varieties of Capitalism literature (Hall & Soskice, 2001), it has been argued that different institutional structures—including industrial relations structures—impact the behaviors of employers in choosing their competitive strategy. Thus, centralized negotiating structures and platforms will help employee representatives negotiate family-friendly arrangements with employers, but also change the way employers behave in choosing their strategies for competition—taking more of a high-performance route. In sum, strength of the trade union, as well as the collective bargaining structures are likely to impact the way companies behave in providing workers with flexitime. Studies have also shown that collective bargaining coverage rates and union density is positively correlated to the use/provision of flexible working arrangements (Berg, Appelbaum, Bailey, & Kalleberg, 2004; Chung, 2009, 2018a; Lyness et al., 2012; Präg & Mills, 2014).

#### Demands/ Culture

It can be expected that countries with a higher proportion of women in the labor market will be those where there are larger demands for family-friendly policies at the company level (Ortega, 2009). This is similar to what is expected at the company level, as will be discussed below. A larger proportion of women in the labor market is expected to change the work culture within organizations to be more family-friendly, because of more demands throughout the labor market regardless of the number of women working in that specific company. Empirical evidence supports this, and use of family-friendly and flexible working arrangements have been shown to be positively related to female labor market participation rates (Chung, 2009, 2014; Ortega, 2009; Präg & Mills, 2014), although others have shown that there are no significant relationships once affluence of the country is taken into account (Lyness et al., 2012). Similarly, normative views on women’s role in the market and household may also influence the way employers provide flexible work arrangements. In countries where gender norms are positive toward women and especially mothers working, there may be more demand from workers toward employers to provide family-friendly arrangements (Kassinis & Stavrou, 2013; Lyness & Judiesch, 2008).

Using Mincer’s (1962) theory of the relationship between affluence and people’s preference toward leisure over paid work time, Präg and Mills (2014) argue that greater affluence of a country will influence worker’s willingness to work fixed hours. In fact, GDP per capita has been positively linked to the use of flexible working arrangements (Chung, 2014; Lyness et al., 2012; Präg & Mills, 2014), although it has been examined only through individual-level data thus far. Similarly, some studies (Chung, 2014; Den Dulk et al., 2013) directly examine the work culture of the country—namely, work centrality, to see how it can change the company’s provision of flexible working arrangements. It is assumed that in cultures where work is more central to one’s life people are likely to work longer, and companies are not likely to provide various flexible working arrangements. Work centrality cultures have shown to reduce the use of family-friendly arrangements, including flexitime, working-time banking, grouped with right to part-time work and right to reduce working hours. In fact, it has been shown to be one of the most important factors explaining the company’s provision of flexible working arrangements when examining company-level data (Chung, 2014).

#### Economic Conditions/Structures

When the economy is in a stain and there is greater labor supply than demand, this may decrease workers’ negotiation power in use of family-friendly arrangements. On the other hand, when there is greater demand than supply, employers may use family-friendly arrangements as incentives to help recruit and maintain workers (Aryee, Luk, & Stone, 1998; Batt & Valcour, 2003; Chung, 2009; Den Dulk et al., 2013). Prevalence of service sectors and public sectors have also been examined to see the how the structure of the economy as a whole has an influence on individual companies through the diffusion of practices (Chung, 2009, 2014; Lyness et al., 2012; Präg & Mills, 2014). Service sectors and public sectors are more likely to adapt to flexible working arrangements (see the next section). It is thus hypothesized that when these sectors dominate the economy, this may change the work practices of the whole country—thus diffusion of work practices across sectors. The prevalence of the service sector can also be linked to the theory of deindustrialization. Deindustrialization, that is the increase of service sector employment in the economy, has been linked to changes in labor market regulations, public sector employment, as well as general changes in the market structure (Esping-Andersen, 1999; Iversen & Cusack, 2000). Results are mixed and can be found in Table 21.1.

### Company-Level Determinants

In this section, we will go into greater detail about who has access to family-friendly arrangements, specifically focusing on flexible working arrangements—namely, family-friendly working-time arrangements and the ability to work from home. Here, I will use the term flexible working arrangements to discuss this. Before moving on, we need to discuss the different factors that can shape the company’s capacity or willingness to provide flexible working arrangements. Unlike statutory policies, where worker’s access to national-level family-friendly policies is guided by law, and limiting access may come with legal consequences, provision of occupational-level family-friendly policies will largely depend on employers. Many academics (e.g., Dex & Smith, 2002; Seeleib-Kaiser & Fleckenstein, 2009; Wiß, 2017) distinguish between structural and agency factors in explaining which companies provide flexible working or broader family-friendly policies. Structural factors are factors that prohibit or enable companies to provide flexible working and other family-friendly arrangements. For example, company size and sector are some key structural factors. Due to the administrative costs that are involved in providing these arrangements, larger companies may find it easier to administer and may have more resources to provide it. Having said that, small- and medium-sized companies may be able to provide more informal or ad hoc arrangements (Dex & Scheibl, 2001). The type of work that is being done has always been noted as one of the biggest constraints to the introduction flexible work arrangements by managers (Van Wanrooy et al., 2013). There are jobs where it is harder to apply flexible working arrangements than in others due to, for example, production structure (machinery, clients demand, etc.) or sensitivities toward certain business cycles. This would mean that certain jobs in sectors such as manufacturing, construction, education, retail, and health and social services may be restricted in their application of flexible working arrangements. Public sector employers, on the other hand, have been seen to be better at providing flexible working and other types of family-friendly arrangements because they are not as sensitive to business cycles (Evans, 2001).

Agency factors pertain more to the willingness of managers and/or the push they get from workers to provide family-friendly/flexible working policies. Agency factors include a range of factors including the composition of workers as well as the existence of (strong) unions, and/or characteristics of managers. For example, scholars have noted that theoretically more women in the company would mean that there will be a higher demand for, and thus higher prevalence of, family-friendly arrangements within that company (Goodstein, 1994). However, empirically, at least in the case of flexible working arrangements, this is not the case (Adler, 1993; Chung, 2019b; Glass & Estes, 1997). This may be because employers are more reluctant to trust women, especially mothers, to privilege work above care/housework (Williams et al., 2013), and believe that women may abuse their ability to work flexibly to essentially do less work. Based on the power resource theory (Korpi, 1989) powerful unions may drive employers to provide schedule control to their workers as a part of their efforts to improve working conditions. What is more, organized labor within the establishment might allow for the introduction of family-friendly policies that managers would not have adopted (Seeleib-Kaiser & Fleckenstein, 2009). In this case, unionized workplaces with employee representatives should be the ones where family-friendly flexible work arrangements will be most prevalent. Empirically, however, the results are rather mixed—some saying that unions matter in the provision and access to flexible working arrangements (e.g., Berg, Kossek, Misra, & Belman, 2014; Seeleib-Kaiser & Fleckenstein, 2009), others noting that there is no significant effect (e.g., Chung, 2018a, 2019a), and some noting that this depends on the country (e.g., Wiß, 2017). On the other hand, some studies argue that rather than unions, managers are important in the introduction of family policies at the company level. For example, companies with supportive managers will be more likely to provide workers with family-friendly flexible work arrangements (Hammer, Kossek, Yragui, Bodner, & Hanson, 2009; Kossek, Hammer, Kelly, & Moen, 2014; Minnotte, Cook, & Minnotte, 2010) and are places where workers feel like they are more able to take up the arrangements (Cooper & Baird, 2015). Some studies argue that female managers are more likely to provide family-friendly arrangements to their workers (Galinsky & Bond, 1998; Ingram & Simons, 1995), however, recent studies have shown no significant association between having a female manager and workers’ access to flexible and other types of family-friendly arrangements (Chung, 2018a, 2019a).

### Individual-Level Determinants

Now we look more closely at individuals’ access to family-friendly arrangements, again specifically focusing on flexible working arrangements. To better understand what can explain who has access to flexible working arrangements, we need to understand the dual nature of flexible working arrangements. Flexible working arrangements are not only used to meet the demands of workers—in particular working parents within the company—but also used to enhance performance outcomes of the company (Brescoll, Glass, & Sedlovskaya, 2013; Den Dulk et al., 2013; Ortega, 2009; Osterman, 1995). High-performance or high-involvement strategy scholars argue that when workers have more control or discretion over their work, this will increase their performance outcomes (Appelbaum, Bailey, Berg, Kalleberg, & Bailey, 2000; Davis & Kalleberg, 2006). Flexible working can be seen as a part of this high-performance strategy specifically aimed at enhancing the performance and productivity of workers.

When understanding the dual nature of flexible and other family-friendly working arrangements (Rapoport, Bailyn, Fletcher, & Pruitt, 2002), we can think of three distinctive principles employers can use to decide who gets access to family-friendly/flexible working arrangements; namely, principle of need, equity, and equality (see also, Lambert & Haley-Lock, 2004; Swanberg, Pitt-Catsouphes, & Drescher-Burke, 2005). When employers are genuinely interested in addressing the work–family needs of workers, those with the most family demands or most need of family-friendly arrangements are likely to request and use flexible work arrangements (Golden, 2009). In addition, companies with workers with more family responsibilities are likely to face a higher demand to provide family-friendly arrangements (Goodstein, 1994), explaining why some studies—especially looking at company-level data, and manager’s perceived provision—have linked the proportion of female workers in a company to the likelihood of the company providing flexible working arrangements (Bardoel, Moss, Smyrnios, & Tharenou, 1999; Dex & Smith, 2002; Kerkhofs, Chung, & Ester, 2008; Wood, De Menezes, & Lasaosa, 2003). However, other studies—especially when looking at individual-level data and workers’ perceived access—have shown that unlike expectation, female-dominated workplaces are where workers are less likely to access family-friendly flexible working arrangements (Adler, 1993; Chung, 2019b; Glass, 1990; Glass & Finley, 2002). This may be because rather than responding to the demands for flexible working, employers are more interested in the enhanced performance/outcomes gained from introducing the arrangements—i.e., the so-called principle of equity. When employers’ motivation for providing flexible working arrangements are driven by the principles of equity, companies will provide these arrangements only to workers they can reap benefit out of - e.g., workers who managers think will work harder or will increase their productivity when working flexibly. In this case, we can expect it to be used more in knowledge-intensive fields (Brescoll et al., 2013) and provided to workers with higher occupational statuses/skills levels in expectation that it will enhance their productivity. This is why many studies have shown that high-skilled workers and workers in higher occupational groups are more likely to gain access to family-friendly arrangements (Chung, 2019a; Gerstel & Clawson, 2014; Glass, 1990; Golden, 2009, 2001; Kelly & Kalev, 2006; Nagar, 2002; Ortega, 2009; Wiß, 2017). Some scholars (Adler, 1993; Schieman, Milkie, & Galvin, 2009) also argue that especially flexible working, where workers gain more control over when and where they carry out their work, is given to higher status workers—again those who are valued in the organization and most likely higher skilled, and possibly in a better bargaining position. On the other hand, workers in disadvantaged positions—e.g., low wage, low-skilled, lower educated—are least likely to have such access (e.g., Golden, 2009; Swanberg et al., 2005; Wiß, 2017). Chung (2018a) examines the degree of access “outsiders” (Schwander & Häusermann, 2013)—workers in disadvantaged/weak positions within the labor market—have to family-friendly/flexible working-time arrangements across Europe. What she finds is that although fixed-term contract status does not influence one’s access to flexible working arrangements (unlike what was found in previous studies, Präg & Mills, 2014), low skilled and those who perceive their jobs to be insecure were significantly less likely to feel that they had access to flexible working arrangements. She concludes that workers’ relative bargaining power may be highly relevant in explaining one’s access to family-friendly/flexible working arrangements.

Lastly, scholars (Lambert & Haley-Lock, 2004; Swanberg et al., 2005) also argue that some companies may implement the principle of equality when providing family-friendly arrangements. In this case, access to arrangements will be provided to all workers equally, regardless of their care demands or potential performance outcome. There is no evidence of this based on empirical studies.

## Outcomes of Family-Friendly Arrangements/Flexible Working

###  Performance Outcomes

There is a wealth of studies that have been done around the so-called “ business case” for flexible working and family-friendly arrangements and performance outcomes (for an overview, see Beauregard & Henry, 2009; De Menezes & Kelliher, 2011; Kelliher & De Menezes, 2019). To sum these studies up, flexible working arrangements and other types of family-friendly arrangements have been shown to have positive links to increasing workers’ organizational commitment, job satisfaction, loyalty, and reduced turnover intention (see also, Masuda et al., 2012; Moen et al., 2017; Ruppanner, Lee, & Huffman, 2018). In turn, the provision of these arrangements is linked to increased worker retention, and reduced worker recruitment problems (Aryee et al., 1998; Kerkhofs et al., 2008; Kossek & Ollier-Malaterre, 2019). In addition, flexible working has been linked to reduced sickness, absenteeism, health, and other undesirable well-being outcomes (see also, Avendano & Panico, 2017; Moen et al., 2016). Some studies have also tried to link worker productivity/organizational performance directly with family-friendly arrangements, for example, such as profit and return on investment, labor productivity, etc. (Chung, 2009; De Menezes & Kelliher, 2011).

### Work–Life Balance

In relation to work–family conflict and work–life balance the evidence is mixed—especially when examining the relationship between work–family conflict and flexible working arrangements. Although some studies show that flexible working reduces work–family conflict for workers (Kelly et al., 2014), others show that the impact is rather minimal (Allen, Johnson, Kiburz, & Shockley, 2013; Michel, Kotrba, Mitchelson, Clark, & Baltes, 2011). Some argue that, especially working from home, may actually increase work–family conflict (Chung, 2017; Duxbury, Higgins, & Lee, 1994; Golden, Veiga, & Simsek, 2006). This may largely depend on the organizational contexts (Van der Lippe & Lippényi, 2018) and national contexts (Lott, 2015). In other words, in more family-friendly contexts flexible working arrangements are more likely to lead to better work–life balance outcomes.

One main reason why flexible working arrangements do not improve work–life balance of workers, or even increase work–family conflict, is because flexible working can lead to workers working longer overtime in paid work or to work spilling over to family spheres. A number of recent studies have shown how flexible working can result in workers working harder and/or longer hours, in many cases (unpaid) overtime hours (Bathini & Kandathil, 2019; Chung & Van der Horst, 2018a; Glass & Noonan, 2016; Kelliher & Anderson, 2010; Lott & Chung, 2016; Schieman & Young, 2010). Other studies have shown that flexible working can lead to mental spillover of work, of workers worrying or thinking about work when not at work (Lott, 2018).

### Flexible Working and Gender Inequality

The extent to which flexible working leads to increased working hours/work intensity is not the same for men and women. Men are more likely to increase their (unpaid) overtime hours when working flexibly (Chung & Van der Horst, 2018a; Glass & Noonan, 2016; Lott & Chung, 2016). This is largely due to the social normative views about gender roles between heterosexual couples. Although there are some changes, men still do and are expected to take on the breadwinning role especially after childbirth (Knight & Brinton, 2017; Miani & Hoorens, 2014; Scott & Clery, 2013) and women are expected to—and actually do—carry out the bulk of caregiving as well as housework (Bianchi, Sayer, Milkie, & Robinson, 2012; Dotti Sani & Treas, 2016; Hochschild & Machung, 1989; Hook, 2006; Scott & Clery, 2013). Such gendered divisions of labor and social normative views about mothers’ and fathers’ roles shape the outcomes of flexible working (Chung & Van der Lippe, 2018). While men increase their working hours, women on the other hand increase their childcare/housework hours when working flexibly (Hilbrecht, Shaw, Johnson, & Andrey, 2013; Kim, 2018; Kurowska, 2018; Lott, 2019; Radcliffe & Cassell, 2014; Sullivan & Lewis, 2001). Clawson and Gerstel (2014) argues that, in this way, flexible working allows workers—especially middle-class workers—to “do gender” (West & Zimmerman, 1987). In other words, flexible working may “allow” workers to adhere to the social normative gender roles prescribed within societies, thus traditionalizing gender roles (Chung & Van der Lippe, 2018). However, it should be noted that this gendered outcome of flexible working is not inevitable. Kurowska (2018), in her analysis comparing Sweden and Poland, shows how in countries where more egalitarian gender norms prevail, men and women may have more similar outcomes when working flexibly. Again, the gender context matters.

Such gendered outcomes of flexible working also impact people’s perceptions toward flexible workers. For example, qualitative studies have shown that when women take up flexible working, for example, working from home, those around them expect women to carry out domestic work simultaneously while working (Hilbrecht et al., 2013; Shaw, Andrey, & Johnson, 2003; Sullivan & Lewis, 2001). This then feeds into the extent to which workers are likely to gain access to flexible working arrangements. Studies have shown that women, especially mothers, are less likely to gain access to flexible working arrangements, even when not used for care purposes (Brescoll et al., 2013; Munsch, 2016). This can explain why flexible working arrangements that provide workers more control over their work are less likely to be provided in female-dominated workplaces—see sections above. What is more, such preconceived notions of where workers’ priorities lie and how they will use flexible working arrangements will naturally shape what the consequences of flexible working for one’s career. Lott and Chung (2016) show how women are unlikely to gain financial premiums as their male counterparts do, even when they work similar levels of overtime. Williams et al. (2013) speak of the flexibility stigma. This is the stigma and the negative career consequences workers using family-friendly arrangements face, largely due to the fact that such take-up makes them deviate from the ideal worker image. Here an ideal worker is that of a worker who does not have any other responsibilities outside of work, and privileges work above everything else (Acker, 1990; Williams, 1999). Although there is a dispute on whether men may face a double stigma of “femininity stigma” (Rudman & Mescher, 2013) in that they deviate away from the ideal worker image alongside the image of masculine breadwinner roles, evidence suggests that this is not the case (Chung, 2018b; Coltrane, Miller, DeHaan, & Stewart, 2013).

In this sense, flexible working can potentially increase gender inequalities in the labor market, due to the preconceived notion people will make about women’s flexible working. However, the picture is much more complex. Several studies have shown that flexible working may allow women to work longer hours than they would have otherwise after childbirth (Chung & Van der Horst, 2018b). In other words, flexible working—especially workers’ ability to control when and where they work—may reduce their need to go into part-time jobs. Part-time jobs in most cases entail occupational downgrading (Connolly & Gregory, 2008, 2009), resulting in career penalties/income loss across the life course and considered one of the key causes of the persistent gender pay gap (Costa Dias, Robert, & Parodi, 2018). Thus, giving workers more control to meet family demands may help workers maintain their careers. Similar results have been found by several scholars. Flexible working—again workers’ control over their work—has been shown to help women stay in relatively stressful yet high paying occupations (Fuller & Hirsh, 2018), and workplaces with flexible working arrangements are those where the gender wage gap is smaller (Van der Lippe, Van Breeschoten, & Van Hek, 2018). In this sense, we need more evidence to see how these rather conflicting directions of impact of flexible working on the gender pay gap act in the longer term for workers.

## Conclusion and Future Research Agenda

As we have seen in this chapter, there has been a great rise in the demands for more family-friendly and specifically flexible working arrangements by workers. Studies have shown that flexible working, and many other family-friendly arrangements, are not necessarily provided to address work–life balance demands of workers but also used to enhance performance outcomes. This can explain why many have found that it is mostly the high-skilled workers in higher statuses that gain access to these arrangements—a topic also addressed by Begall and Van der Lippe in Chapter 10.1007/978-3-030-54618-2_22 in this volume. The chapter also raised issues around the discrepancies between provision stated at the company or state level, versus workers’ access to flexible and other family-friendly policies. Such real access to arrangements is shaped by workers’ individual (and collective) negotiation/bargaining power, and prevalence of flexibility stigma or the fear of negative career consequences when taking up family-friendly arrangements. More studies need to be done to find out how to ensure that all workers with the demand for more flexible working and other family-friendly arrangements can get access to them, without fear of repercussion.

The chapter further examined the role of national context factors in shaping companies’ provision of and workers’ access to flexible and other family-friendly arrangements. There is increasing evidence to show that there is a positive rather than a negative relationship between generous national-level policies and generous family-friendly policies at the company level/better access to family-friendly policies from the workers’ perceptive, pointing to a “crowding in” effect. However, more needs to be examined in terms of the dynamics in which this effect takes place, as well as whether there are variations across different types of family policies as well as across different groups of the population. Further research is needed to examine whether national family policy contexts shape the outcome of flexible and other family-friendly working practices.

Similarly, the chapter summarized some of the key outcomes of flexible and other family-friendly arrangements. As the review has shown the relationship is not as clear-cut as expected, with flexible working leading to worse rather than better work–life balance outcomes, and gender inequality outcomes in some cases. More research needs to be done to examine these relationships further. Especially of interest for many policy makers and company managers will be the impact of flexible working in the longer run for gender equality. The chapter has shown how there are rather conflicting dynamics at play—on one hand enabling better access to labor market participation for women, but on the other, enabling or enforcing traditional divisions of labor between men and women. More could be explored in terms of what can be done to ensure to avoid some of these negative unintended consequences of flexible working.

Finally, we need more data to capture the extent to which family-friendly policies are being provided at the company level. As seen from this chapter, the most recent cross-national comparative data comes from 2004, more than a decade and a half from the publication of this chapter. More recent data are presented in Chapter 10.1007/978-3-030-54618-2_22 in this volume by Begall and Van der Lippe. A large number of companies provide family-friendly policies above and beyond the national regulations for a number of reasons including skilled worker recruitment and maintenance, as well as to enhance the corporate social responsibility image. On the other hand, we know from case studies that many companies do not even allow workers access to national-level provisions that are supposed to be protected by law, may it be due to lack of knowledge or lack of any bargaining power. Company-level surveys that can capture both managers’ and workers’ perspectives on a wide range of family-friendly company-level arrangements, above and beyond flexible working arrangements, are needed to fully understand workers’ true access to family policies.

## Footnotes

1 See this link: https://eur-lex.europa.eu/legal-content/EN/TXT/?uri=CELEX%3A52017PC0253.

2 This section is adapted from Chung, H. (2014) Explaining the provision of flexitime in companies across Europe (in the pre- and post-crisis Europe): role of national contexts. WAF working paper 1. Canterbury: University of Kent.
